# Supplementary material for: Perturbations in the microbiota-gut-brain axis shaped by social status loss
Source: Commun Biol. 2025 Mar 8;8:401. doi: 10.1038/s42003-025-07850-1 (PMC11890786; doi:10.1038/s42003-025-07850-1)
Supplement: Supplementary file 1 — Supplementary Figs. [file 42003_2025_7850_MOESM1_ESM.pdf]

Supplementary Figure 1

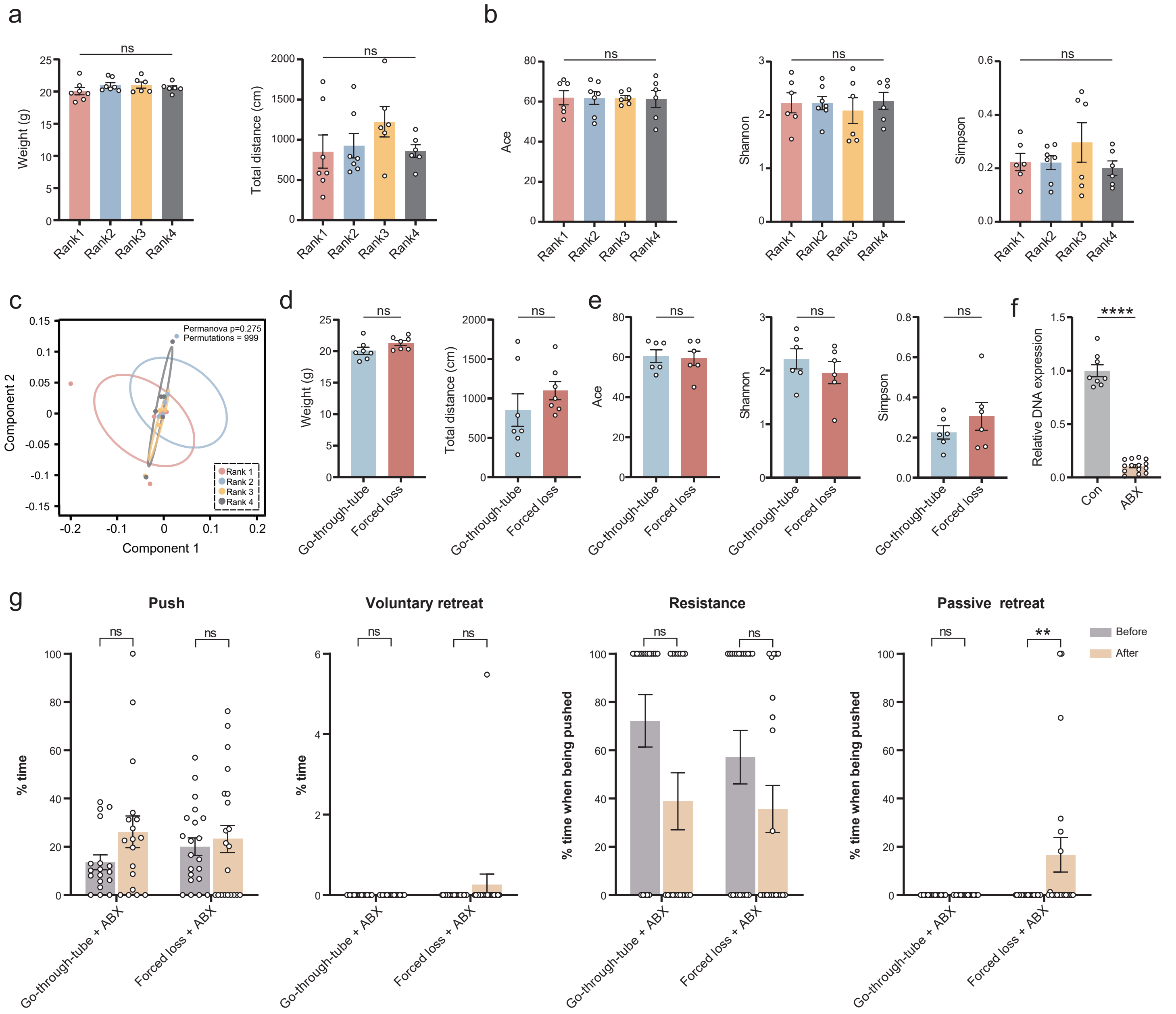

Fig. S1: Changes in the diversity, richness, and composition of the gut microbiota in mice.

a Body weights and total distances in the open field tests for mice with different ranks (Rank1, n = 7; Rank2, n = 7; Rank3, n = 6; Rank4, n = 6; Rank1 vs Rank2 vs Rank3 vs Rank4, no statistical difference in p values; data are mean ± SEM; Kruskal-Wallis test, two-tailed)

b Alpha diversity indices of gut microbiota in mice with different ranks (Rank1, n = 6; Rank2, n = 7; Rank3, n = 6; Rank4, n = 6; Rank1 vs Rank2 vs Rank3 vs Rank4, no statistical difference in p values; data are mean ± SEM; Wilcoxon rank-sum test, two-tailed).

c The overall characteristics of gut microbiota displayed using PCoA based on the abund_jaccard distance, with internal differences analyzed by PERMANOVA test (permutations = 999, p = 0.275).

d Body weights and total distances in the open field tests for mice in the go-through-tube and forced loss groups (Go-through-tube, n = 7; Forced loss, n = 7; Go-through-tube vs. Forced loss, no statistical difference in p values; data are mean ± SEM; T test, two-tailed).

e Alpha diversity indices of the gut microbiota in go-through-tube mice and forced loss mice (Go-through-tube, n = 6; Forced loss, n = 6; Go-through-tube vs. Forced loss, no statistical difference in p values; data are mean ± SEM; Wilcoxon rank-sum test, two-tailed).

f DNA expression of gut microbiota genes in the control and ABX groups (Con, n = 8; ABX, n = 14; Con vs. ABX, **** $p < 0.0001$; data are mean ± SEM; T test, two-tailed).

g Time percentages of pushing, voluntary retreating, resistance (when pushed by an opponent), and passive retreating (when pushed by an opponent) during tube test for microbiota-depleted mice before and after go-through-tube or forced loss procedure (Go-through-tube + ABX, n = 6 mice, 18 trials; forced loss + ABX , n = 7 mice, 21 trials. *$p < 0.05$, **$p < 0.01$, ***$p < 0.001$, ****$p < 0.0001$, ns, not significant; data are mean ± SEM; Mann-Whitney test, two-tailed).

Supplementary Figure 2

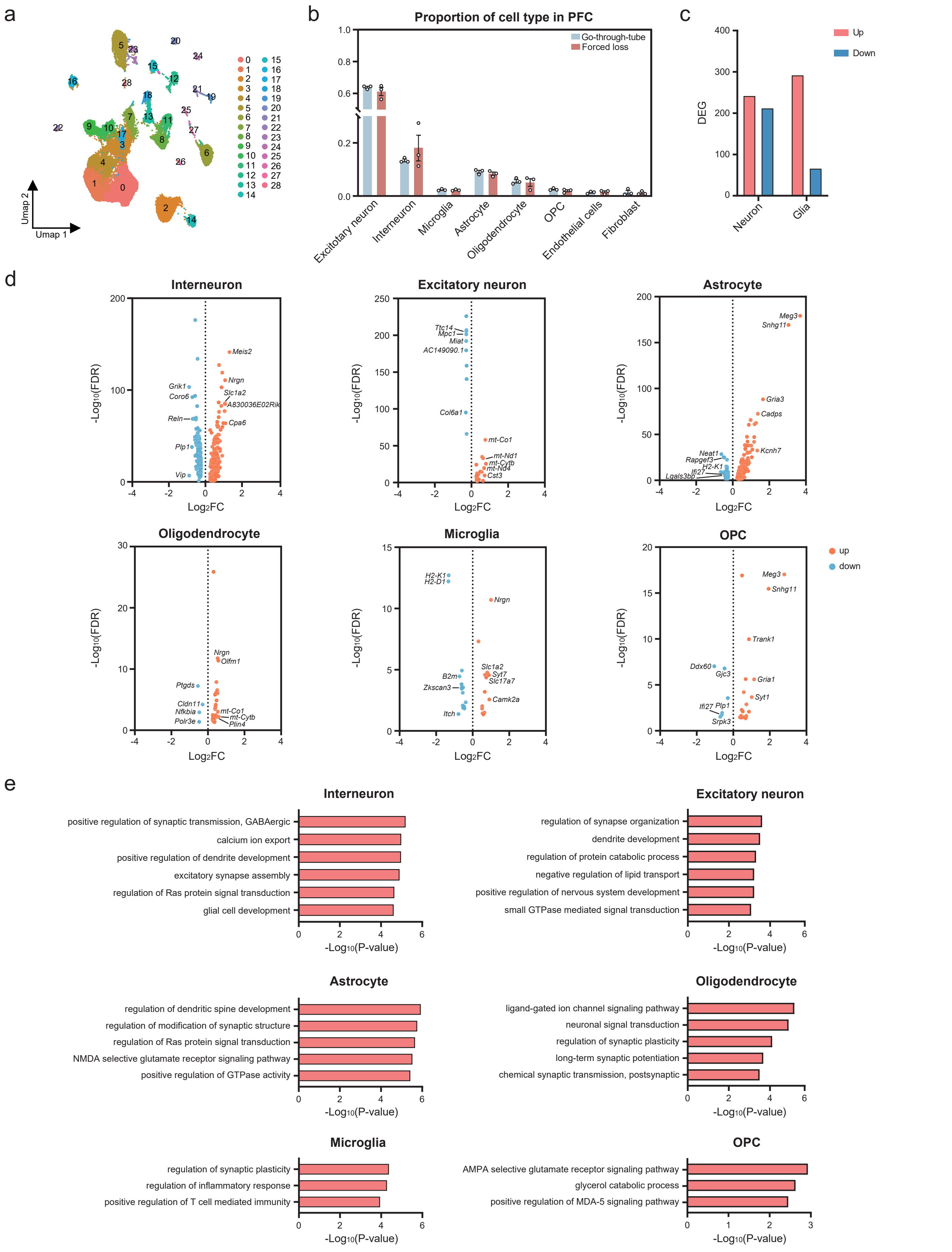

Fig. S2: DEGs and the functions of major cell types in the PFC.
a The UMAP graph showed single-cell dimensionality reduction clusters in PFC. The UMAP graph showed that 52626 nuclei were clustered in PFC to obtain 29 pretreated cell clusters.
b Proportion of cell types in PFC (Go-through-tube, n = 3; Forced loss, n = 3; Go-through-tube vs. Forced loss, no statistical difference in p values; data are mean ± SEM; Mann-Whitney test, two-tailed).
c The amount of DEG up-regulated and down-regulated in neurons and glial cells.
d Volcano plots showed DEGs expressed in six major cell types in PFC (log_2FC > 0.25 or < -0.25, and FDR < 0.05).
e GO enrichment analysis showed the functions of DEGs enrichment expressed in interneurons, excitatory neurons, astrocytes, oligodendrocytes, microglia and OPC (p < 0.05).

Supplementary Figure 3

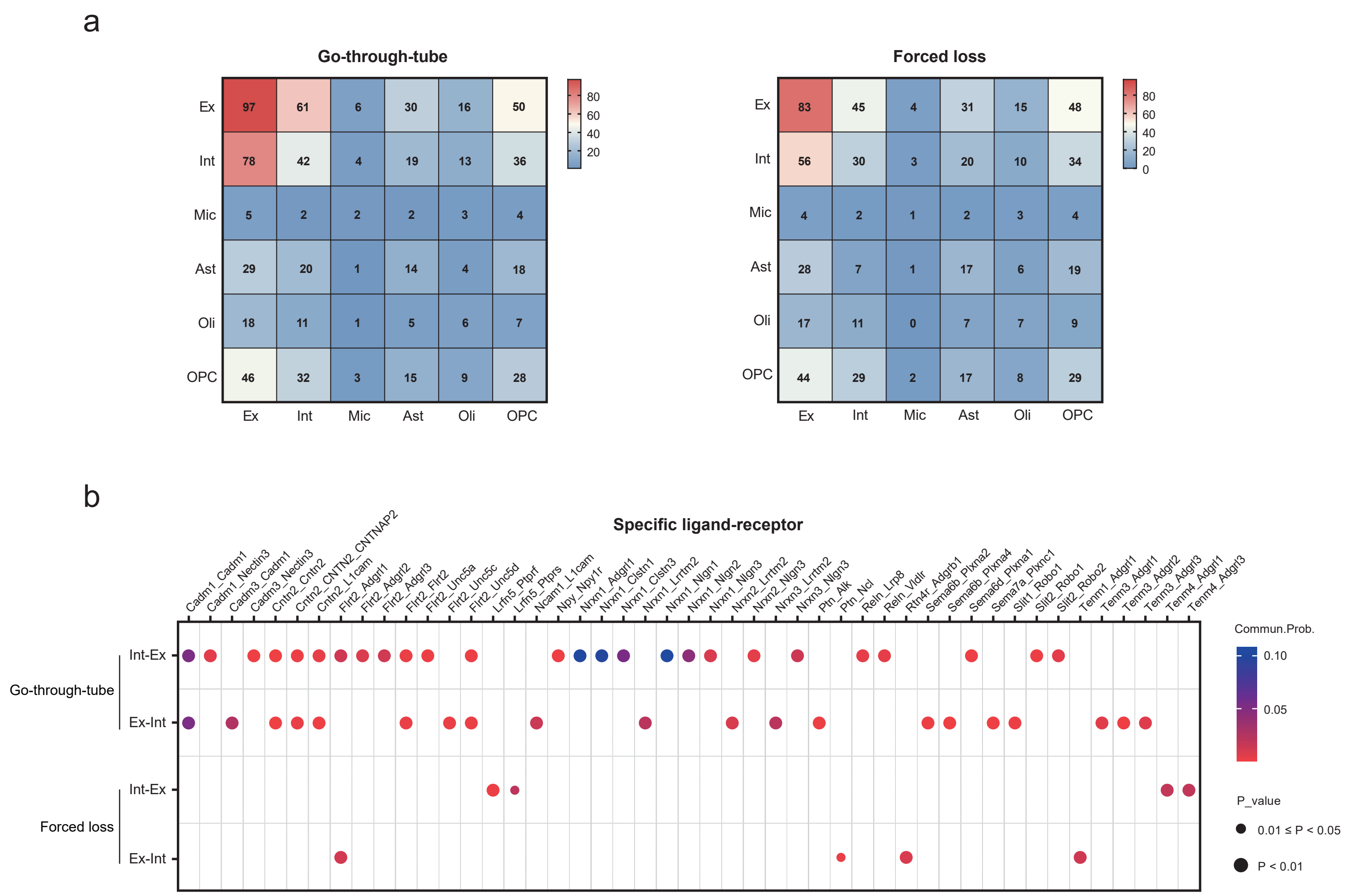

Fig. S3: The number and specific function of ligand-receptor pairs in the go-through-tube and forced loss groups.
a The number of all activated ligand-receptor pairs in the go-through-tube and forced loss groups (Go-through-tube, $n = 3$; Forced loss, $n = 3$).
b Functions of specific connections in the ligand-receptor relationships between interneurons and excitatory neurons ($p < 0.05$).

Supplementary Figure 4

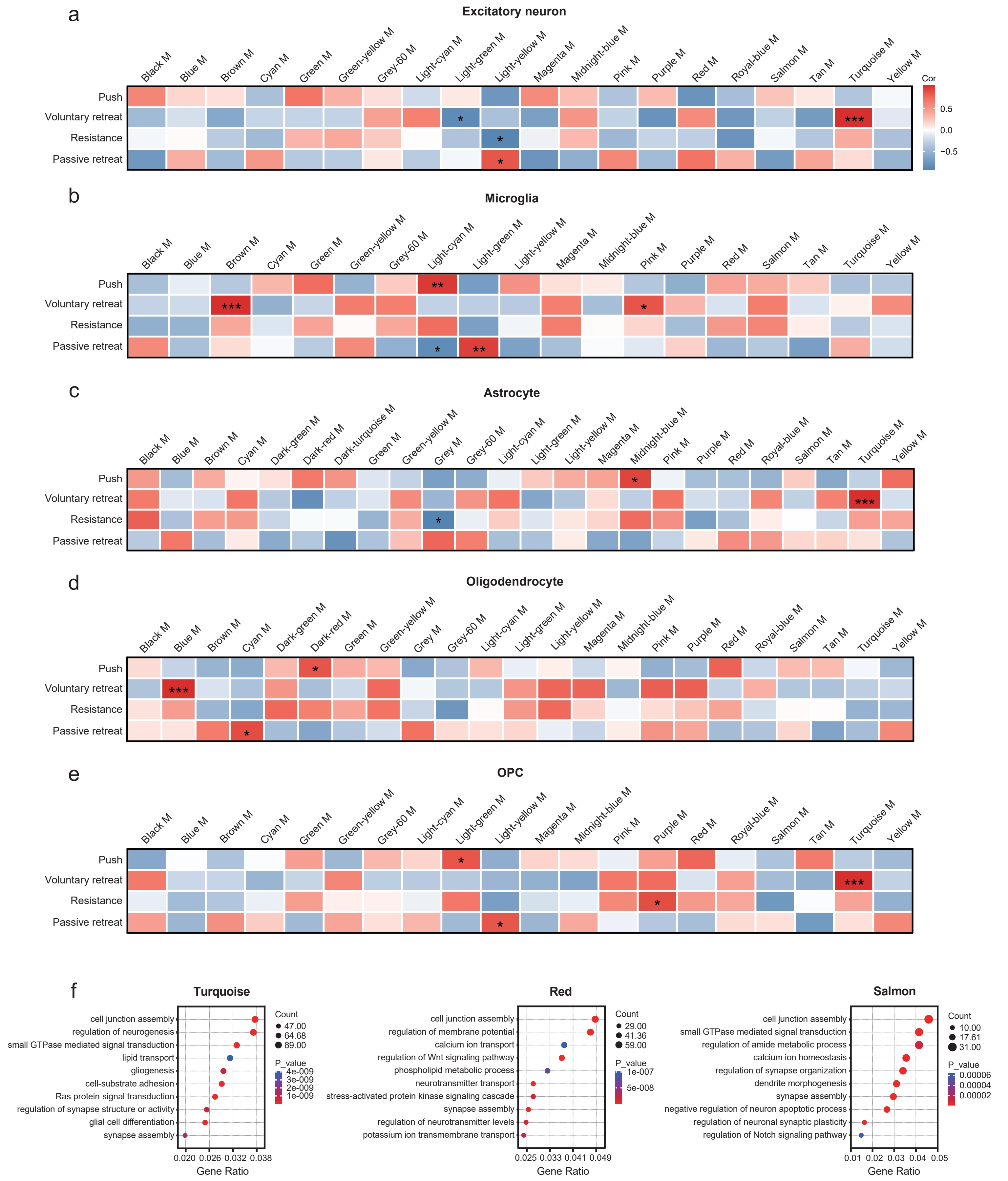

Fig. S4: Correlation between gene modules of differential cell types and forced loss related behaviors.
a-e The heatmap showed gene modules associated with four behaviors (push, voluntary retreat, resistance, passive retreat) in excitatory neurons, microglia, astrocytes, oligodendrocytes and OPC ($r > 0.6$ or < -0.6, *$p < 0.05$, **$p < 0.01$, ***$p < 0.001$, two-tailed Pearson correlation).
f GO enrichment analysis showed the functions and pathways of genes enrichment in three modules (turquoise, red, salmon) associated with voluntary retreat in interneurons ($p < 0.05$).
