# Supplementary material for: Perturbations in the microbiota-gut-brain axis shaped by social status loss
Source: Commun Biol. 2025 Mar 8;8:401. doi: 10.1038/s42003-025-07850-1 (PMC11890786; doi:10.1038/s42003-025-07850-1)
Supplement: Supplementary file 4 — Description of Supplementary Materials [file 42003_2025_7850_MOESM4_ESM.pdf]

Description of Additional Supplementary Files

File name: Supplementary Data 1

Description: The source data for all Figures

File name: Supplementary Data 2

Description: The source data for all Supplementary Figures
