# Supplementary material for: Perturbations in the microbiota-gut-brain axis shaped by social status loss
Source: Commun Biol. 2025 Mar 8;8:401. doi: 10.1038/s42003-025-07850-1 (PMC11890786; doi:10.1038/s42003-025-07850-1)
Supplement: Supplementary file 5 — Reporting summary [file 42003_2025_7850_MOESM5_ESM.pdf]

nature portfolio

Corresponding author(s): Peng Zheng, Jing Wu

Last updated by author(s): Feb 26, 2025

Reporting Summary

Nature Portfolio wishes to improve the reproducibility of the work that we publish. This form provides structure for consistency and transparency in reporting. For further information on Nature Portfolio policies, see our Editorial Policies and the Editorial Policy Checklist.

Statistics

For all statistical analyses, confirm that the following items are present in the figure legend, table legend, main text, or Methods section.

n/a	Confirmed	
☐	☒	The exact sample size (*n*) for each experimental group/condition, given as a discrete number and unit of measurement
☐	☒	A statement on whether measurements were taken from distinct samples or whether the same sample was measured repeatedly
☐	☒	The statistical test(s) used AND whether they are one- or two-sided<br>*Only common tests should be described solely by name; describe more complex techniques in the Methods section.*
☐	☒	A description of all covariates tested
☐	☒	A description of any assumptions or corrections, such as tests of normality and adjustment for multiple comparisons
☐	☒	A full description of the statistical parameters including central tendency (e.g. means) or other basic estimates (e.g. regression coefficient) AND variation (e.g. standard deviation) or associated estimates of uncertainty (e.g. confidence intervals)
☐	☒	For null hypothesis testing, the test statistic (e.g. F, t, r) with confidence intervals, effect sizes, degrees of freedom and P value noted<br>*Give P values as exact values whenever suitable.*
☒	☐	For Bayesian analysis, information on the choice of priors and Markov chain Monte Carlo settings
☒	☐	For hierarchical and complex designs, identification of the appropriate level for tests and full reporting of outcomes
☐	☒	Estimates of effect sizes (e.g. Cohen's *d*, Pearson's *r*), indicating how they were calculated

Our web collection on statistics for biologists contains articles on many of the points above.

Software and code

Policy information about availability of computer code

Data collection	Sequencing: Illumina NovaSeq 6000
Data analysis	Noldus Observer XT (v13.0); R studio (v4.3.3); Seurat (v5.0.3); CeleScope (v1.5.2); STAR (v2.6.1b); FeatureCounts (v2.0.1); Cytoscape (v3.10.2); WGCNA (v1.72); GraphPad Prism software (v9.5.0); Origin (v2024); CellChat package (v2.1.2)

For manuscripts utilizing custom algorithms or software that are central to the research but not yet described in published literature, software must be made available to editors and reviewers. We strongly encourage code deposition in a community repository (e.g. GitHub). See the Nature Portfolio guidelines for submitting code & software for further information.

Data

Policy information about availability of data

All manuscripts must include a data availability statement. This statement should provide the following information, where applicable:

- Accession codes, unique identifiers, or web links for publicly available datasets
- A description of any restrictions on data availability
- For clinical datasets or third party data, please ensure that the statement adheres to our policy

All data supporting the findings of this study are available within the paper and its supplementary information.

Research involving human participants, their data, or biological material

Policy information about studies with human participants or human data. See also policy information about sex, gender (identity/presentation), and sexual orientation and race, ethnicity and racism.

Reporting on sex and gender	N/A
Reporting on race, ethnicity, or other socially relevant groupings	N/A
Population characteristics	N/A
Recruitment	N/A
Ethics oversight	N/A

Note that full information on the approval of the study protocol must also be provided in the manuscript.

Field-specific reporting

Please select the one below that is the best fit for your research. If you are not sure, read the appropriate sections before making your selection.

☒ Life sciences ☐ Behavioural & social sciences ☐ Ecological, evolutionary & environmental sciences

For a reference copy of the document with all sections, see nature.com/documents/nr-reporting-summary-flat.pdf

Life sciences study design

All studies must disclose on these points even when the disclosure is negative.

Sample size	There are a total of 46 mouse samples. For the 16S rRNA-seq analysis and metagenomic sequencing, 6 samples are from the go-through-tube group and 6 from the force loss group. The ranks were distributed as follows: Rank 1 have 6 mice, Rank 2 have 7 mice, Rank 3 have 6 mice, and Rank 4 have 6 mice. The microbiota-depleted mice were divided into go-through-tube group (n = 6) and force loss group (n = 7). For transcriptomic analysis, 7 samples were from the go-through-tube group and 7 from the forced loss group. Single-cell sequencing samples included 3 mice from the go-through-tube group and 3 from the forced loss group.
Data exclusions	No data were excluded.
Replication	For the mice experiment, we denoted the experimental points as 'n'-values, serving as biological replicates, in each figure legend. All experiments performed are reproducible across biological and technical replicates.
Randomization	The go-through-tube group and the forced loss group were randomly assigned. The grouping of mice into different ranks was determined by the results of the tube test.
Blinding	All behavioral videos were independently checked by 2 observers blinded to the social status assignment. The experimenter was blinded to the allocation information until completion of statistical analyses. To avoid bias, all samples were treated equally with the same rigorous criteria.

Reporting for specific materials, systems and methods

We require information from authors about some types of materials, experimental systems and methods used in many studies. Here, indicate whether each material, system or method listed is relevant to your study. If you are not sure if a list item applies to your research, read the appropriate section before selecting a response.

Materials & experimental systems

n/a	Involved in the study	
☒	☐	Antibodies
☒	☐	Eukaryotic cell lines
☒	☐	Palaeontology and archaeology
☐	☒	Animals and other organisms
☒	☐	Clinical data
☒	☐	Dual use research of concern
☒	☐	Plants

Methods

n/a	Involved in the study	
☒	☐	ChIP-seq
☒	☐	Flow cytometry
☒	☐	MRI-based neuroimaging

Animals and other research organisms

Policy information about studies involving animals; ARRIVE guidelines recommended for reporting animal research, and Sex and Gender in Research

Laboratory animals	C57BL/6J mice, male, 6-8 weeks old
Wild animals	NO wild animals involved in this study
Reporting on sex	Only males were included in this study
Field-collected samples	NO field-collected samples involved in this study
Ethics oversight	The animal experimental procedures were conducted in accordance with the Guidance for the Care of Laboratory Animals and approved by the Ethics Committee of Chongqing Medical University (Chongqing, China).

Note that full information on the approval of the study protocol must also be provided in the manuscript.

Plants

Seed stocks	N/A
Novel plant genotypes	N/A
Authentication	N/A
